# Applications, Limitations, and Considerations of Clinical Trials in a Dish

**DOI:** 10.3390/bioengineering11111096

**Published:** 2024-10-30

**Authors:** Amatullah Mir, Angie Zhu, Rico Lau, Nicolás Barr, Zyva Sheikh, Diana Acuna, Anuhya Dayal, Narutoshi Hibino

**Affiliations:** 1 Section of Cardiac Surgery, Department of Surgery, University of Chicago, 5841 S. Maryland Ave., Chicago, IL 60637, USA; amir1@uchicago.edu (A.M.); angiez@uchicago.edu (A.Z.); rico1604@uchicago.edu (R.L.); nab1@uchicago.edu (N.B.); zyvasheikh@uchicago.edu (Z.S.); dacuna1@uchicago.edu (D.A.); t-9adaya@uchicago.edu (A.D.); 2 Pediatric Cardiac Surgery, Advocate Children’s Hospital, 4440 W 95th St., Oak Lawn, IL 60453, USA

**Keywords:** iPSCs, organ-on-a-chip, clinical trials in dish, bioprinting, tissue engineering, vasculature, 3D modeling, scaffolds, spheroids, 3D printing, vascularization, hard tissue, stem cells, biomechanics, 3D-printed sensors, bioinks, 3D tissues, soft tissue

## Abstract

Recent advancements in biotechnology forged the path for clinical trials in dish (CTiDs) to advance as a popular method of experimentation in biomedicine. CTiDs play a fundamental role in translational research through technologies such as induced pluripotent stem cells, whole genome sequencing, and organs-on-a-chip. In this review, we explore advancements that enable these CTiD biotechnologies and their applications in animal testing, disease modeling, and space radiation technologies. Furthermore, this review dissects the advantages and disadvantages of CTiDs, as well as their regulatory considerations. Lastly, we evaluate the challenges that CTiDs pose and the role of CTiDs in future experimentation.

## 1. Introduction

Clinical trials in a dish (CTiDs) play an integral role in translational research, serving as the key assessment and analysis of the feasibility, costs, risks, and benefits of applying these discoveries in the clinical setting. That being said, clinical trials often incur countless obstacles, such as financial costs, length of human studies, and gathering of a sufficient patient population. Alternative outlooks to clinical trials need to be considered to keep up with rapidly advancing medical research, to ensure that resources, time, and money for clinical trials are being allocated optimally, and to quicken and improve the assessment strength of clinical trials [1].

**Figure 1:**
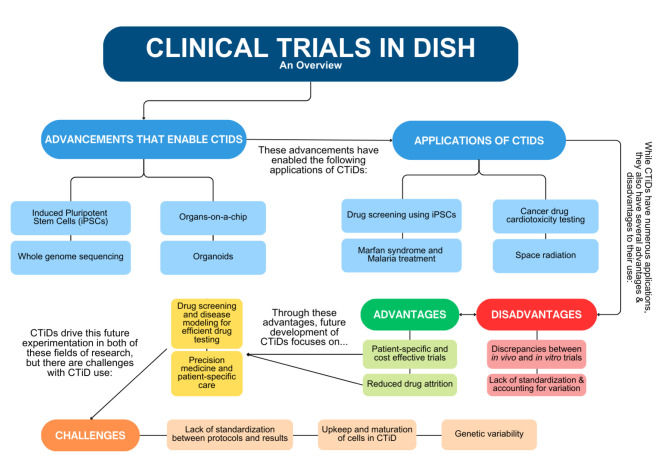
Roadmap overview of “Applications, Limitations, and Considerations for Clinical Trials in Dish” [2].

This comprehensive review (Figure 1) will first explain the biotechnologies behind CTiDs, then analyze the applications of CTiDs already being implemented in current research, assess the advantages and disadvantages of this approach to translational research, and finally discuss the future initiatives and challenges with CTiDs (Figure 1). The experimentation of clinical trials in a dish are ongoing and rapidly developing. The progression of clinical trials in a dish provides opportunities for drug discovery, toxicity testing modeling, and personalized medicine applications.

## 2. Biotechnology Advancements Enabling CTiDs

The development of CTiDs would not have been possible without several other biotechnology advancements, such as whole genome sequencing, induced pluripotent stem cells (iPSCs), and organs-on-a-chip (OOCs). Each of these biological advancements have provided the foundation for CTiDs to successfully recreate the in vivo environment of target patients, maintaining the specific genetic details of diseases and encouraging in vitro interactions between different cell types.

### 2.1. Induced Pluripotent Stem Cells (iPSCs)

**Figure 2:**
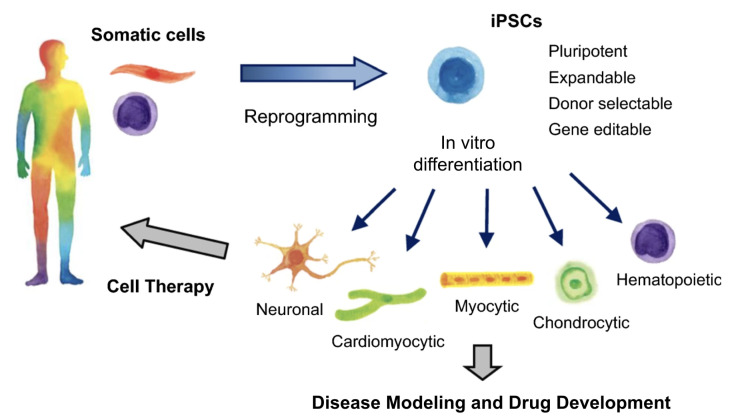
Applications of iPSCs in disease modeling, drug development, and cell therapy. Adapted from Sugimoto et al. [2].

Induced pluripotent stem cells (iPSCs) are a type of pluripotent stem cell derived from adult somatic cells and are genetically reprogrammed into an embryonic stem cell-like state [3]. The regenerative capacity of iPSCs is crucial to CTiDs, as they can differentiate into a variety of functional cell types in vitro, such as cardiomyocytes, endothelial cells, and smooth muscle cells [3]. iPSCs are often patient-specific, which means these cells contain the genetic information of the disease found in the patient that they came from. This is particularly advantageous when wanting to utilize a human model to understand the mechanisms behind certain diseases or the outcome of pharmaceutical drugs (Figure 2).

Harnessing the impressive potential of iPSCs to recapitulate in vivo environments allows for their ultimate use in CTiDs. Drug candidates can be tested on many iPSCs from different people with the same clinical syndrome but potentially different underlying causes to identify the subset of responders versus nonresponders [4]. This information about patients and their response phenotype can be used to enroll patients in eventual in vivo clinical trials, thus improving the chances of the drug’s efficacy [4]. The use of iPSCs in CTiDs allows for the identification of the most suitable medication for a particular patient without requiring the patient to undergo any adverse effects.

### 2.2. Whole Genome Sequencing (alt. Genome Editing Technology)

In combination with iPSCs, recent advances in genome editing technology have accelerated the creation of CTiDs. The acquisition of patient samples with the desired disease-causing mutation is unpredictable, iPSC generation is time-consuming, and some iPSC disease models lack an isogenic unaffected control [5]. To address these issues, genome editing can be used to directly introduce specific genetic mutations into the iPSCs to make disease models, with the unedited cells serving as an isogenic control [5]. Wang et al. demonstrate the use of zinc finger nuclease technology to overexpress the dominant negative mutation associated with long QT syndrome (LQTS), and they used these genetically edited cells to generate LQTS models that can be used for drug screening. While both genome-edited iPSC models and patient-derived iPSC models can be used for drug screening, genome-editing removes the additional step of recruiting patients and increases the certainty of having cells with the desired genotype [5].

### 2.3. Organs-on-a-Chip (OOCs)

**Figure 3:**
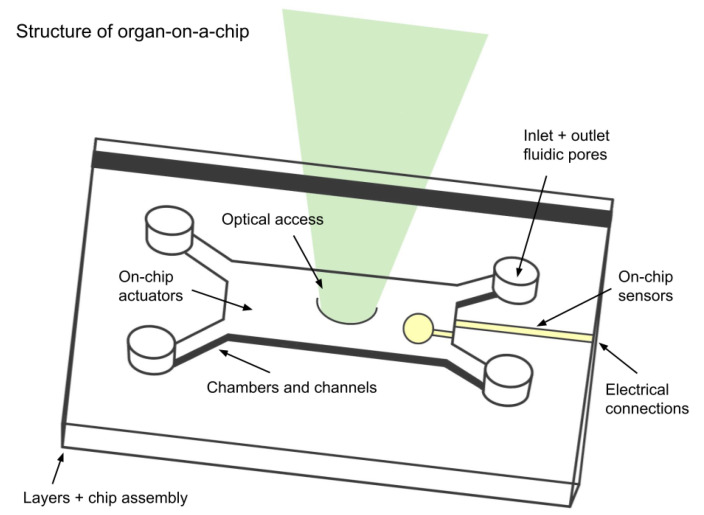
Structure of the organ-on-a-chip device. Adapted from Kurth et al. [7].

Organs-on-a-chip (OOCs) are microfluidic devices that mimic the structure and function of specific organs and tissues (Figure 3).

They serve a similar purpose to CTiDs, in that they are also used to enhance preclinical development of drugs by testing on an in vitro replica of an organ-specific in vivo environment. OOCs offer the advantage of creating a platform for iPSCs that incorporates the three-dimensional structure of tissues. Before CTiDs, OOCs demonstrated the necessity to extend beyond isolated 2D cell cultures and animal models and to establish an in vitro system that captures the complexities of human physiology in order to holistically assess drug testing models [6].

### 2.4. Organoids

**Figure 4:**
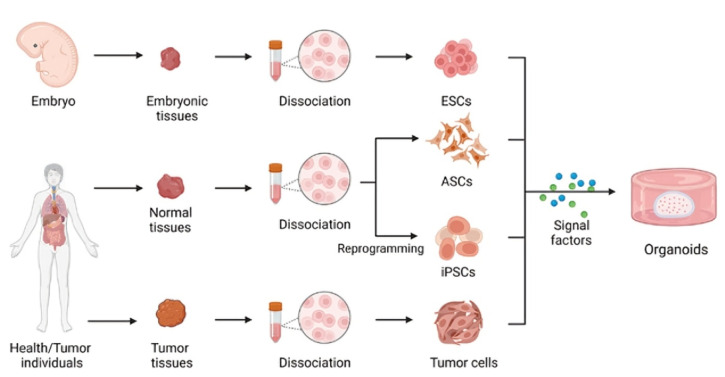
Methods of organoid development in vitro. Cell sources for organoids include embryonic stem cells, adult stem cells, tumor cells, and induced pluripotent stem cells. Adapted from [8].

Three-dimensional structures that are formed from stem cells and self-arrange to develop organ tissue are called organoids. Organoids are simple to generate [8] (Figure 4); they may be derived from single cells or multiple adult or pluripotent stem cells [9]. Organoids mimic physiological conditions and parental genetic stability, hence are optimal options for drug testing and disease modeling. Organoids also have a faster growth and high culture success rate. While organoids pose several advantages, the technology is not yet mature. The growth of organoids is not consistent, and the technology has been explored by a limited number of laboratories [10].

Here is a summary of the biotechnology advancements that enable CTiD (Table 1).

## 3. Applications of Clinical Trials in a Dish

There are numerous applications of CTiDs, namely in drug development, cancer drug cardiotoxicity testing, and space radiation applications.

### 3.1. hiPSCs and CTiDs in Drug Development

hiPSCs are becoming increasingly popular as the primary foundation for the entire preclinical drug development pipeline due to their numerous advantages. First, hiPSCs are patient-specific and, therefore, generate a disease phenotype depending on the selected individuals. To this end, deriving a line from an unaffected relative will provide an excellent control to compare the effects of certain drugs, minimizing genetic variability. Second, the scalability of hiPSCs conceptually allows for the infinite generation of any human cell type. However, in practice, in vitro differentiation protocols have only been generated for a small group of specific cell types, i.e., neurons, cardiomyocytes, keratinocytes, hepatocytes, and more [13]. Nevertheless, the potential for boundless generation of patient-specific cells remains. With this, high-throughput drug screening assays analyzing toxicity, efficacy, discovery, and drug development in general, can be repeatedly performed. Such assays are also applicable to early-onset diseases and the early stages of disease, a characteristic unique to hiPSCs. Prior to their discovery, drug testing was typically tested in relevant patient populations only after phase II clinical trials, which also resulted in a higher drug attrition rate [13]. With the newer hiPSC model, patient samples are involved in the very beginning, preventing the potential waste of resources and ultimately reducing the cost of drug development. This unlocks access to data at the earliest stages of drug development and throughout the drug discovery process. More specifically, the potential to identify likely deregulated pathways resulting from disease prior to a clinical trial now exists. Implementing high-throughput assays within a large cohort of patients can select individuals sensitive to a drug for testing, further reducing the cost and improving efficiency for clinical trials. These advantages may also be applicable to combinations of drugs and other therapies. For example, a phase 1 clinical trial was recently conducted by the FDA to investigate the potential for late sodium and calcium current blocking drugs as remedies for drug-induced long QT syndrome [14]. Following this, iPSC-cardiomyocytes were generated from the clinical trial participants to examine the efficacy of patient-specific cells in generating similar results [15].

In addition to the advantages of hiPSCs and CTiDs as described above, the compatibility of modern day biotechnology, namely whole-exome sequencing, gene editing, and RNAi, with patient-derived hiPSCs presents CTiD as a powerful tool. Whole-exome sequencing identifies mutations of interest and variants involved in the presentation of a specific disease. This provides an initial idea of the expected disease severity dependent on the present genetic risk variants. Such variants can subsequently be introduced into iPSC lines to further understand their roles in modulating the disease [16]. In addition, cutting-edge gene editing tools can be applied to hiPSC lines to control disease phenotypes. Whether one intends to correct mutations to produce otherwise healthy control iPSC lines or to generate disease models without patient involvement, gene editing tools allow for creative genetic modification of patient-specific cell lines. Moreover, RNAi technology produces the opportunity to identify disease-relevant biological signatures for clarifying disease mechanisms and discovering novel drug targets [13]. Specifically, the resulting phenotype of an RNAi knockdown of gene candidates can act as a complementary model to compare therapeutics that mimic such a knockdown in iPSC-derived disease models.

### 3.2. CTiDs in Replacing Animal Testing

Since the discovery of iPSCs, animal testing is gradually being overshadowed in the world of drug testing. However, this is not without good reason. Animal models are poor predictors of clinical trials, given the physiological differences between animals and humans: the resting heart rate in mice is 500–700 bpm, about 10 times more rapid than in humans [17]. On the other hand, hiPSC-cardiomyocytes would show exceedingly more similar physiological and pharmacological responses to various degrees. Given the genetic differences, the translation of results in animals to humans is quite poor. Habashi et al. found that both the neutralization of TGF-B with an antibody and a blockade of angiotensin II receptor type 1 (AT1R) with losartan rescues the disease phenotype [18]. To this end, a series of clinical trials were run in human patients comparing losartan to B-blockers. They found that losartan generated no statistical improvement in children and young adults when compared to B-blockers [19], a result inconsistent with the findings in mice. This sequence of investigations exacerbates the need for an alternative to animal testing, and encourages the use of hiPSCs and clinical trials in a dish for future drug development.

### 3.3. CTiD in Genetics: Marfan Syndrome and Malaria

Marfan syndrome (MFS) is a disease resulting from a mutation in Fibrillin-1 (FBN1), a key connective tissue ECM protein. A disease phenotype typically includes a weakened aortic wall and stiffened vessels. Davaapil and colleagues differentiated VSMCs from patient-derived iPSCs to reiterate specific core aspects of the vascular disease in patients, such as the fragmentation of ECM microfibrils and matrix metalloproteinase expression [16,20]. It was found that exposing cells to cyclic mechanical stretch further magnified the disease phenotype, indicative of mechanosensing abnormalities and agreeing with current notions regarding the disease progression mechanism. To this end, the disease phenotypes were rescued with CRISPR-Cas9-mediated single nucleotide modification, an example of the use of gene editing with CTiDs. In another study, host–pathogen interactions between Plasmodium infections and liver cells were extensively studied using iPSC-derived hepatocyte-like cells (iHLCs) [21]. It was shown that these iHLCs can be robustly genetically modified, enabling future investigation into the impact of host genetics in liver-stage malaria.

### 3.4. CTiD in Cancer Drug Cardiotoxicity Testing

Cardiovascular disease (CVD) is the global leading cause of death. Being that the cardiovascular complications of cancer therapy are a significant contributor to CVD, the development of novel preclinical testing tools to assess the safety and efficacy of therapies is undoubtedly a paramount issue. To this end, hiPSCs and CTiDs show excellent promise as a means to analyze drug-induced cardiotoxicity. Isolated adult cardiomyocytes (CMs) are unavailable for long-term in vitro studies due to their tendency for rapid dedifferentiation. Compared to isolated adult cardiomyocytes, hiPSC-cardiomyocytes (hiPSC-CMs) are readily producible, more robust, and effective for both short and long-term cellular responses. In other words, hiPSC-CMs are effective in defining acute versus chronic cardiotoxicity, and the intercalations between both. This flexibility allows for the identification of early markers of cardiac injury, which in turn help predict longer-term cardiotoxicity. For example, it may be impossible to detect long-term effects in vitro for most cancers, but with hiPSC-CMs, early signs and sensitive upstream biomarkers (i.e., transcriptional changes and cardiac troponins) can be detected to guide inferences on cardiotoxic mechanisms. Such in vitro demonstrations with hiPSCs and CTiDs, in corroboration with information provided from toxicological and dose-escalation clinical studies, mean that false-positive safety findings can be avoided, ultimately avoiding the discarding of lifesaving drugs [22].

Two in vitro studies involving doxorubicin, a conventional chemotherapy, exemplified the ability of hiPSC-CMs to detect cardiotoxic effects. Louisse et al. administered low-dose doxorubicin on hiPSC-CMs and conducted phenotypic assays on day 14. They found significant mitochondrial dysfunction, i.e., depolarized membrane potentials and elevated calcium levels, after two weeks of doxorubicin dosage [23]. Holmgren et al. combined hiPSC-CMs and protein, mRNA, and microRNA data to identify multiple cardiotoxicity mechanisms and suggestive putative biomarkers for chemotherapy-induced cardiotoxicity [24].

### 3.5. CTiD in Space Radiation

The use of hiPSCs and CTiDs in space radiation biology is still in its infancy stage. Conventional cell models used prior to the implementation of hiPSCs were primary cell types, for example, fibroblasts. However, such primary cell types lack genetic diversity and physiologically relevant environments. They nevertheless are an accessible robust means for investigating the biology behind human responses to space radiation. A pioneering example of hiPSC usage in this field is when Becker and colleagues exposed differentiated hiPSC-CMs to 5 and 10 Gy of X-rays. These irradiated hiPSC-CMs were then characterized with RNA-sequencing after 48 h. The data revealed that radiation decreased the beating rate of CMs, and higher doses of X-rays were more likely to change the electrophysiological spatial distribution [25]. Another example includes when Wnorowski flew hiPSC-CMs onboard the ISS in collaboration with NASA for a 5.5 week time period. The resulting hiPSC-CMs displayed altered calcium handling and dysregulated genes involved in mitochondrial metabolism [25]. These two examples not only exemplify the potential for discovering space radiation countermeasures through the means of hiPSCs and CTiDs, but also the boundless potential of these strategies for applications even in the seemingly nichest of fields in biology.

### 3.6. More Applications of CTiDs

With the continuously evolving nature of CTiDs, the aforementioned applications are only a few of the countless implementations the scientific community has come up with. An additional application of CTiDs and hiPSCs relates to regenerative medicine, more specifically, the implementation of these strategies in creating tissue-engineered vascular grafts (TEVGs). Differentiated iPSC-CMs are an excellent platform for generating TEVGs, as the patient-specificity of these cardiomyocytes offer biocompatibility and immune-compatibility to the vascular constructs. iPSC-CMs are particularly useful in pediatric patients to align the growth of these cardiovascular grafts with normal growth and development [16]. A final cardiac application of CTiDs and hiPSCs involves aligned coaxial PCL-gelatin nanofiber cardiac patches. Kumar and colleagues designed such cardiac patches with mechanical and biomimetic properties, generating a potential drug screening platform for drug development and cardiotoxicity studies. hiPSC-CMs cultured on these patches exhibited synchronous contractions, exhibited a rod-shaped morphology, and reacted quickly to cardiac drugs [26].

Clinical trials in a dish are, however, hardly limited to cardiology. iPSCs were proven to effectively model host–pathogen interactions between hepatitis B and hepatocytes. In both donor hepatocytes and iPS-hepatocyte-like cells, hepatitis B infection was shown to be enhanced by blocking interferon-stimulated gene induction [27]. In another study, iPSCs were differentiated into forebrain-specific human neural progenitor cells (hNPCs) to screen for potential antiviral drugs against the Zika virus, which is known to efficiently target hNPCs. The study identified 116 out of 6000 compounds that suppress Zika virus replication and activity [28].

**Figure 5:**
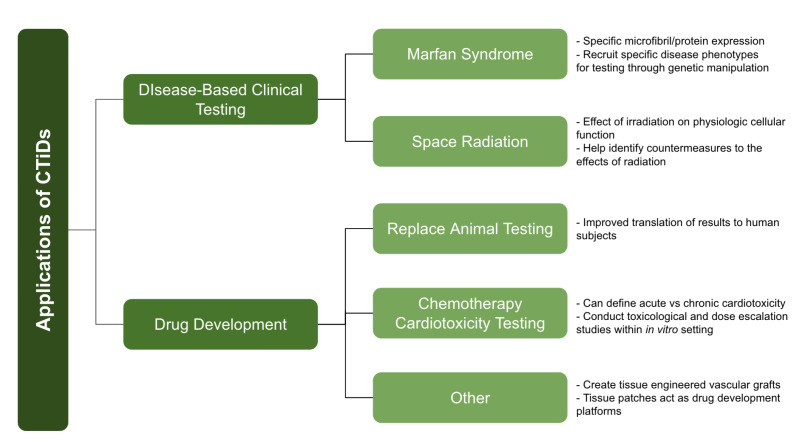
Applications of CTiDs (summarized).

Here is a summary of CTiD applications and their advancements in science (Figure 5, Table 2).

## 4. Advantages and Disadvantages of In Vitro Clinical Trials

Clinical trials in a dish bring to the table new opportunities and challenges. Compared to in vivo studies, in vitro studies allow for faster, cheaper, and more patient-specific trials. CTiDs also hold the key to reducing drug attrition, a growing concern in the rapidly expanding medical industry. However, with these advantages, unique challenges follow as well. CTiDs currently lack standardization and a means to account for variability. Moreover, considering the complex landscape of stem cell-based studies, uncertainties in molecular mechanisms arise.

### 4.1. Cheaper and Quicker Trials with Patient Specificity

With the advent of medical discovery, naturally, comes an increase in price. The cost of clinical trials specifically continue to skyrocket as new drugs are discovered. A majority of the cost stems from personnel associated items—trail conduction, clinical regulation, patients with a rare disease. A study looking at the average cost of clinical trials found that the median cost was 3.4 million dollars for phase I, 8.6 for phase II, and 21.4 million for phase III [30]. Aside from cost, Lasagna’s law also reflects difficulties in patient recruitment and retention as a clinical study progresses [31].

CTiDs introduce a method to both help reduce these costs and alleviate the innate challenges of clinical trials. With in vitro studies, one sample from a donor can quintessentially be used indefinitely. This way, new patients do not need to be recruited and maintained, drastically reducing the price. Moreover, once the sample has been acquired, all remaining manipulations are strictly cell-based, meaning patient organization and retention are no longer an obstacle [32]. The lengthy nature of clinical trials, as patients are followed over time, is also reduced in CTiDs as cellular manipulations and experiments tend to occur on a shorter time scale.

CTiDs also provide new utility. Using a patient’s tissue samples, CTiDs provide patient specificity, something clinical trials lack. In one study, scientists demonstrated that human-induced pluripotent stem cell-derived cardiomyocytes (hiPSC-CMs), tissue samples collected for CTiD, help predict the cardiomyocyte of doxorubicin to breast cancer patients, and that the reactivity was specific to conditions each patient had experienced [22]. In another study, scientists found that an hiPSC-CM model successfully recapitulated specific patient sensitivity to the drug moxifloxacin [33]. These studies demonstrate CTiD’s unique advantage; by using samples from the patient, patient-specific effects can be studied. Such specificity greatly expands the breadth of research. Prior to the use of CTiDs, it was difficult to study diverse ethnic groups due to research limitations and cost. Clinical research was largely based on and tailored to the representative majority. Using CTiDs with small groups, ailments that affect smaller groups can be better understood and eventually treated [32]. The same applies for rare diseases, or even individuals.

### 4.2. Reduction of Drug Attrition Before Clinical Testing Results in Increased Safety

One issue the medical field faces is the increase in drug attrition in clinical development. Clinical trials are often lengthy and tedious, but many tested drugs fail, leading to wasted resources and money. CTiD has great potential to reduce such drug attrition. Its properties allow it to be a valuable resource to investigate toxicity and predicting clinical safety. Using an in vitro model, otherwise complex organ-specific drug toxicities can be better studied and understood, shedding light on the drug’s feasibility preclinically. On the more simple end, simple cell death can reflect drug toxicity to help narrow drug candidates. On the more complex end, changes in cellular function, like energy metabolism, can be investigated [34]. In one study, researchers used an in vitro monolayer of human-induced pluripotent stem cell-derived cardiomyocytes (IPSC-CMs) and a drug array to calculate the drug’s proarrhythmic potential, predicting drug toxicity even before it was introduced to clinical trials [35]. While a CTiD may not be able to fully predict a drug’s clinical effects, it can help divert money and resources to the most promising drug candidates. Investigating toxicology before in vivo trials also reduces the risk involved for the patient. CTiDs can eliminate toxic drugs immediately, narrowing the range of drugs to the most promising ones and enhancing safety for clinical trial subjects.

### 4.3. In Vivo and In Vitro Discrepancies Due to Molecular Complexities

Some of the disadvantages of a CTiD stem from the cellular basis that comprises it. Biological mechanisms are naturally complex, and these mechanisms are the foundation on which in vitro studies are based. It is difficult to unquestionably accept the results of a CTiD without entirely understanding the underlying mechanisms [36]. It is often difficult to conclude if results observed through a CTiD are scientifically supported or if they are due to variation in scientific methods. Small changes in methods—such as passing technique, cell origin or line, and even passage number—can lead to varying and inconsistent results [36]. Cellular mechanisms are complex, and, as such, CTiDs are biologically complex too.

Another concern that arises from this complexity is the difficulty to fully replicate in vivo conditions in a dish. Using stem cells, which often compromise the cells of CTiDs, as an example, research has recently elucidated the stem cell niche. This niche refers to the system of molecule signals, regulatory networks, nutrient pathways, and so forth, that surround stem cells in the body. Recent engineering efforts have attempted to recreate this niche artificially, and, while great progress has been made [37], no method perfectly recapitulates the niche. As such, with current technology, it is difficult to fully replicate in vivo conditions in vitro.

### 4.4. Standardization and Accounting for Variability

Apart from biological complexities, there also exists regulatory disadvantages of CTiDs. As CTiDs are still relatively new, there is currently no standardized process of assessing quality for stem cell-based in vitro studies. Clinical study methods are heavily regulated, but, in comparison, CTiD conduction lacks such systematized guidelines [36]. This introduces discrepancies and inconsistencies across different studies using CTiDs. Likewise, variability is difficult to assess in CTiDs. Most studies are created with a single hiPSC-derived cell line. This makes the assumption that this specific donor’s cell line is representative of all cell lines and at the center of the population. It is unlikely that one donor or cell line is the perfect average of the sample, and this assumption can lead to misleading conclusions [36].

## 5. Regulatory Considerations for Clinical Trials in a Dish

### 5.1. Importance of New Regulatory Paradigms

The current process for drug research and development involves lengthy and expensive regulatory paradigms, which rarely lead to approval by the US Food and Drug Administration (FDA). The FDA began to require clinical trials and preclinical trials of toxicity for novel drugs in 1961, but the only toxicity paradigms approved were in animal studies [38]. In the past 25 years, only 9.5% of drugs that have passed preclinical trials and entered into the first phase of clinical trials have actually reached the final step of approval by the FDA [38]. Animal models are relatively expensive, low throughput, and often present ethical concerns. Moreover, animal models tend to show controversial results in toxicity when continued on human clinical trials [15].

The FDA has recognized the need for new clinical trial designs and methods to expedite and improve the drug process. In August of 2018, the FDA launched the Complex Innovative Designs Pilot Meeting Program. This program allowed scientists from drug and biologic companies to meet with an FDA agent staff to discuss their novel complex innovative design. The FDA agent staff could then provide intensive feedback on the proposed design to promote future implementation. These innovative clinical trial designs would also be shared with the broader scientific community through presentations to bring to effect clinical trials with reliable methods of assessing drug safety and efficacy [38].

### 5.2. Implementing and Developing Clinical Trials in a Dish Methodologies in Research Studies

CTiDs provide an alternative to animal models for drug testing. Ko et al. highlighted the growing number of studies using iPSC technology to create hiPSC models of rare human diseases for the testing of various drug compounds [39].

These recent studies have shown that hiPSCs can be used for high-volume drug screening due to their ability to express disease mutations corresponding to a wide variety of diseases. Furthermore, genetic mutations can be introduced to iPSCs to produce a combination of mutations to study rare genetic diseases without having to search for a patient with the corresponding mutations. Finally, iPSCs can also be used to predict patient-specific responses. Burridge et al., in 2016, suggested that iPSC-derived cardiomyocytes can predict the specific doxorubicin-induced cardiotoxicity response of an individual at the single cell level, where before, determining patient cardiotoxicity was not possible [22]. Therefore, CTiDs serve as a regulatory paradigm for drug testing, disease modeling, and precision medicine. [15].

The FDA has also sponsored research working to improve current methods in clinical trials and promote in vitro clinical trials. An FDA-sponsored study was performed that validated drug-induced responses on CTiDs by comparing them to the results of Johannesen, in 2015, using a Proarrhythmia Assay to assess the cardiac safety of drug compounds. Johannesen, in 2015, employed electrocardiographic (eCG) biomarkers to predict the risk of drug-induced abnormal heart rhythms. Drug-induced AT syndrome involves a prolongation of the QT interval on an electrocardiogram, putting patients at high risk of ventricular arrhythmias and death [14]. Therefore, drug-induced QT prolongation often results in drugs being removed from the market or prevents potential medicines from reaching the market [14]. The study indicated that late sodium current blocking drugs, such as mexiletine and lidocaine, can shorten drug-induced QT prolongation in human subjects [14]. These results highlight that drugs failing preclinical trials due to cardiotoxicity can be tested when coadministered with late sodium current blockers to mediate the toxic effects.The study also implemented the method of CTiDs through the generation of iPSC-cardiomyocytes from each of the 20 human clinical trial participants. The iPSC-cardiomyocytes were tested by the FDA to determine if the iPSCs had the ability to predict the drug-induced response on the QT interval previously observed [15]. This effort by the FDA will be able to determine the validity of results using iPSCs as determinants for patient-specific response. Furthermore, the iPSCs were generated at two different commercial laboratories to determine if the two produced similar results, suggesting whether the generation of iPSCs can produce constant results. Following the study, the FDA biobanked the iPSCs, promoting additional research in CTiDs [15].

## 6. Future Directions

### 6.1. Efficiency in Drug Development

**Figure 6:**
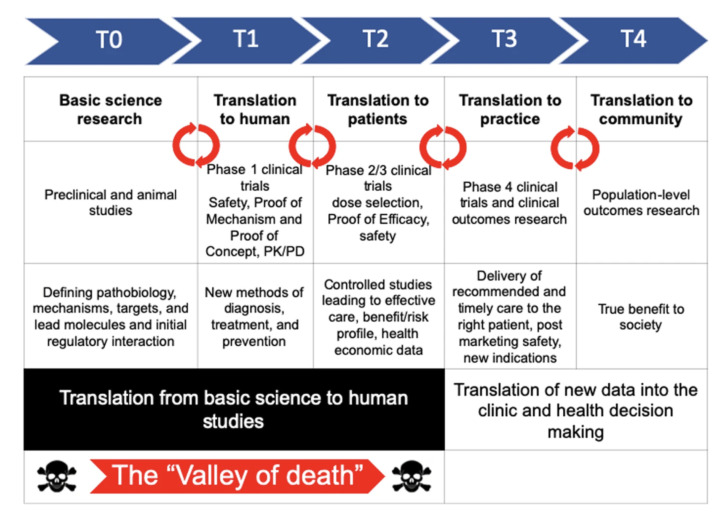
Translation from basic science to human studies. Adapted from Seyhan et al. [1].

The “translational gap” is the difficulty in converting clinical research into practical, industrial applications, which has become a cavernous pit of invested money and effort (Figure 6) [1]. A study from Tufts Center for the Study of Drug Development in 2014 estimated that the cost of producing one drug, including the time value of money, now surpasses $2.6 billion [40]. Of that $2.6 billion, only $200 million is spent in total on the approved drug. The majority of funds are spent on candidate drugs that ultimately fail in testing stages, presenting a large room for improvement. As far as the returns on this translation, approximately 10% of drugs that enter the testing phases are approved fully by the FDA [41]. This low rate of success, combined with the fact that the drug screening process can take up to 12 years, acts as a large road block in the translation from bench to bedside [42].

Novel strategies such as CTiDs may act as bridges to this ever-increasing gap between lab research and clinical trials, which has been coined the “valley of death” [43,44]. CTiDs exist as a path to improve the efficiency at which this largely unsuccessful process takes place. Relative to FDA drug screens, the speed and cost of screening using CTiDs would reduce attrition and investment prices, leading to safer and more abundant drugs on the market. Being notably more flexible than the rigid conditions of standardized human testing, CTiDs bypass difficulties such as the difficulty and invasiveness of tissue collection and achieve a higher accuracy in predicting adverse effects [32,45]. Shelved drugs, which have been erroneously deemed unsafe via false positives in screening, can also be retested with reduced cost and time.

In tandem with drug–cell interactions, drug-on-drug interactions (DDIs) are another realm in which CTiDs are a broader and more efficient strategy. DDIs are the effects of the reaction between two or more drugs in the cell; they are the cause of one-fifth of all adverse drug reactions and are estimated to be the leading cause of 20,000 deaths per year in the United States [46]. DDI detection in a wet lab is inefficient and costly, leading many to use in silico computational methods which may not reflect the cellular environment in its complex totality [47,48]. In vitro assays such as CTiDs are more expedient and would ensure the accurate modeling of DDIs.

### 6.2. Precision Medicine

Screens of drugs rely on testing a microcosm of the larger target population, ensuring an adequately large genetic range to monitor for adverse effects. However, due to differences in allelic frequency between ethnic groups that may be underrepresented, false positives may occur [45]. The construction of a genetic database, which would be implemented by CTiDs, would ensure a greater genetic diversity than current screening methods while still encapsulating the effects of the drug in vitro, as shown by the proof of concept done by Dr. Shinozawa in hiPSC-CMs [33]. Cross-screening between different populations, like the US and Japan for example, is also made more accessible due to the portability of samples [4,49]. Whereas screens often can only encompass drug effects on a macro scale, CTiDs also provide the additional element of patient-specific drug testing. As a hypothetical, it is possible to acquire a cell sample from a patient with an unknown disease and test drugs with greater accuracy and depth before prescription, increasing the chances of a successful and beneficial effect.

## 7. Challenges

### 7.1. Standardization and Validation of Assays

The flexibility of CTiDs provides advantages in the malleability of tissue collection; however, this also causes a problem in the standardization of testing, reprogramming, and differentiation protocols and the validation of results produced and published. Discrepancies between tested cells, which can include the time in culture, passage number, origin of the cell on the body, methods of cellular selection, and general heterogeneity, have lead to inconsistent results across similar tests [50,51,52,53]. Many protocols are specific to the intended cell, making it difficult to standardize them between disciplines. Uniformity is not ideal either, as the usage of a single cell line creates low genetic variability, leading to a screen that will likely not express all adverse drug effects in the targeted population.

With the complexity of iPSC differentiation, all elements of a standard protocol, including but not limited to sourcing, collection, culture, and testing, need to be rigorously followed. Many regulatory guidelines are currently being worked on, as previously discussed. Insofar as protocol standardization, ensuring a dependable and representative collection of donor tissues, the preservation of the integrity of said tissues, and reduction of protocol-induced instability are essential in limiting variability between research projects [32]. Control iPSCs are also required for testing, which raises questions on sourcing and culturing. Genetic differences between sexes and ethnicities mean that no true control cell can possibly be fair in a screen, meaning that controls will need to be screen-specific, likely by inducing mutations [45]. This increases the complexity, time, and cost of screens, although it is necessary for valid results to be obtained.

### 7.2. Cell Upkeep and Maturation

iPSCs have complex culture requirements and multi-step differentiation protocols, often spanning multiple weeks [54,55,56]. The complexity of the cell culture alongside the lengthy time frame and costs of reagents is a potential reason for labs to not wish to integrate CTiDs. Time is a source of potential false negative reads in various drug interactions, as some effects are not detectable for weeks or longer. For example, many neurodegenerative diseases can take decades to manifest, making induction in week-old cells difficult [4]. This may also result in significant latency for patient-specific tests, as the time from tissue collection to significant data may be long. Attempts to speed up this process by skipping the reprogramming phase and going straight from somatic cells to the target differentiated cell, called direct reprogramming, is shown to result in low yields and poor cellular quality [57,58,59].

In addition to challenges in culture, many differentiated cells express a fetal phenotype, not accurately representing the mature phenotype of the cells they are intended to represent. This adds yet another challenge in maturing the cells prior to testing, as immature cells express lower levels of genetic markers, have fetal morphologies, and perform worse on physiological tests. In the case of differentiated cardiac cells, they exhibit poor sarcomeric structure, electrophysiology, and calcium handling [60]. Maturation also worsens the aforementioned issues of complexity, cost, and time. For example, neurological maturation is incredibly slow, further worsening the issue of time for CTiDs [61]. Overall, maturation poses an obstacle for streamlined differentiation for cells to use in CTiDs.

### 7.3. Genetic Variability

**Figure 7:**
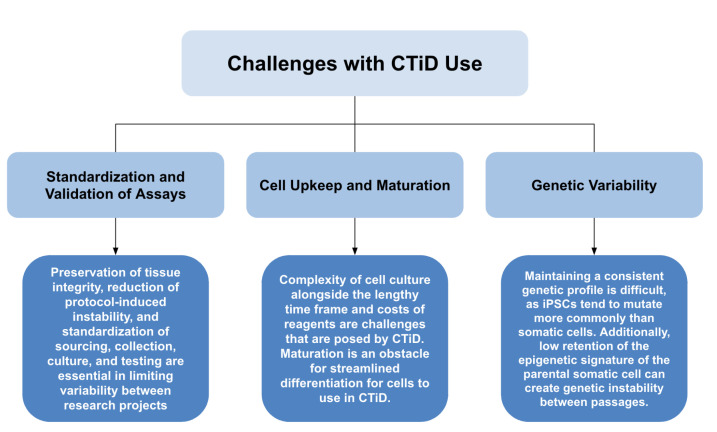
Challenges with CTiD use (summarized).

The genetic diversity of a CTiD assay is important for establishing an adequately large sample to screen for potentially infrequent or rare adverse drug effects. However, a consistent genetic makeup is necessary for ensuring the validity and repeatability of the results. Genetic variability during and between assays is an important factor to consider, as it can limit the effectiveness of the disease modeling and drug treatment. Low retention of the epigenetic signature of the parental somatic cell can create genetic instability between passages, which is compounded by the effects of differentiation-based genetic change [57]. Combined with the fact that iPSCs tend to mutate more commonly than somatic cells, maintaining a consistent genetic profile is difficult [45]. Here is a summary of the challenges of CTiD use and their descriptions (Figure 7).

## 8. Conclusions

CTiDs are an advanced method of analysis of novel drugs, diagnostic assessments, and medical protocols. Driven by biotechnologies such as iPSCs, whole genome sequencing, or organs-on-a-chip, CTiDs illustrate the practical and clinical applicability of these advanced methods. CTiDs have already been integrated in various fields of study, such as for drug development, as a substitute for animal testing, the isolation of specific disease genotypes (such as with Marfan syndrome), cardiotoxicity testing in chemotherapeutic medications, and to study the effects of space on human physiology. CTiDs incur fewer costs, are faster, and are a safer environment for the initial stages of clinical trial testing. However, in vitro experiments will have molecular and structural differences from in vivo subjects, and many CTiDs unsatisfactorily rely on a single human cell line to represent an entire population. Furthermore, this is a relatively new concept without standardization of practice and few regulations. While a powerful technology, both the benefits and deterrents of CTiDs must be considered.

CTiDs allow for the testing of novel medications or procedures in a specific, patient-cell-based environment without the dangers or limitations of working with human subjects. However, the inconsistency of results in experiments among CTiDs, the lack of regulation, and the genetic discrepancies indicate that, ultimately, human clinical trials are still necessary as the final stage of testing. Further research and development of CTiDs will hopefully work to combat these obstacles and enable CTiDs to replace clinical trials in the near future.

## Figures and Tables

**Table 1:** Biotechnology advancements enabling CTiD (summary).

Biotechnology	Description	Advancement	Reference
Induced Pluripotent Stem Cells (iPSCs)	Type of pluripotent stem cell derived from adult somatic cells and are genetically reprogrammed into an embryonic stem cell-like state	Recapitulate in vivo environments using iPSCs, yield personalized treatment options for patients, and enable drug screening + testing.	[11]
Whole Genome Sequencing (alt. Genome Editing Technology)	Technologies that allow for the editing or modifying an organism’s DNA.	Introduces specific genetic mutations in order to model diseases, enables drug screening + testing.	[5]
Organs-on-a-chip (OOCs)	Microfluidic devices that mimic the structure and function of specific organs and tissues	Platforms iPSCs by hosting a 3D structure for tissue growth, allows for experimentation that extends beyond 2D cell culture.	[12]
Organoids	3D structures developed from stem cells and self-organize to form organ-specific tissue	Offers a fast-forming patient-specific organ for drug screening and disease modeling	[8]

**Table 2:** Applications of CTiDs.

Application	Advancement in Science	Reference
Replace Animal Testing	Improved translation of results to human subjects	[17,29]
Marfan Syndrome	Specific microfibril/protein expression, recruit specific disease phenotypes for testing through genetic manipulation	[20]
Chemotherapy + Cardiotoxicity Testing	Can define acute vs. chronic cardiotoxicity, conduct toxicological and dose escalation studies within in vitro setting	[23,24]
Space Radiation	Effect of irradiation on physiologic cellular function, help identify countermeasures to radiations effects	[25]

## Data Availability

The data presented in this study are publicly available as found in the references below.
